# *MADCAM1* Gene Variants as Potential Pharmacogenomics Markers for Vedolizumab Response in Crohn’s Disease Bio-Naïve Patients

**DOI:** 10.3390/diagnostics16162523

**Published:** 2026-08-11

**Authors:** Biljana Stankovic, Vladimir Gasic, Bojan Ristivojevic, Ivana Grubisa, Branka Zukic, Aleksandar Toplicanin, Olgica Latinovic Bosnjak, Srdjan Markovic, Aleksandra Sokic Milutinovic, Sonja Pavlovic

**Affiliations:** 1 Institute of Molecular Genetics and Genetic Engineering, University of Belgrade, 11042 Belgrade, Serbia; biljana.stankovic@imgge.bg.ac.rs (B.S.); vladimir.gasic@imgge.bg.ac.rs (V.G.); bojan.ristivojevic@imgge.bg.ac.rs (B.R.); ivana.grubisa@imgge.bg.ac.rs (I.G.); branka.zukic@imgge.bg.ac.rs (B.Z.); 2 Clinic for Gastroenterohepatology, University Clinical Center of Serbia, 11000 Belgrade, Serbia; aleksandartoplicanin4@gmail.com (A.T.); asokicmilutinovic@gmail.com (A.S.M.); 3 Clinic for Gastroenterology and Hepatology, University Clinical Center of Vojvodina, 21000 Novi Sad, Serbia; olgica.latinovic@gmail.com; 4 School of Medicine, University of Belgrade, 11000 Belgrade, Serbia; srdjanmarkovic@yahoo.com; 5 Department of Gastroenterology and Hepatology, University Hospital Medical Center “Zvezdara”, 11120 Belgrade, Serbia

**Keywords:** Crohn’s disease, vedolizumab, pharmacogenomics, *ITGA4* gene, *ITGB7* gene, *MADCAM1* gene, HLA alleles

## Abstract

**Background/Objectives.** Crohn’s disease (CD) is a chronic immune-mediated inflammatory disease that affects the gastrointestinal tract. The management of CD is complex, and treatment requires numerous therapies. One of the best gut-selective biologic drugs with proven efficacy in induction and maintenance therapy in patients with CD is vedolizumab (VDZ). Nevertheless, not all patients with CD respond to VDZ treatment, possibly due to their individual pharmacogenomic profiles. In this study, the association of variants in genes encoding molecules involved in biological pathways targeted by VDZ, *ITGA4*, *ITGB7*, and *MADCAM1*, with VDZ response was analyzed. In addition, Human Leukocyte Antigen (HLA) alleles were investigated. **Methods.** Whole exome sequencing was performed on 63 CD patients treated with VDZ as first-line biologic therapy. Genetic variants were associated with response to VDZ treatment, estimated as follows: (1) good clinical response (patients assigned to standard maintenance protocol after 14-week induction) or partial (patients assigned to optimized maintenance protocol after 14-week induction); (2) good biochemical response (CRP ≤ 10 mg/L at week 14) or poor (CRP > 10 mg/L at week 14). **Results.** Two *MADCAM1* variants were associated with VDZ response, as defined by CRP values in week 14 of VDZ therapy. The genetic variant *MADCAM1* rs758941486 was associated with poor CRP reduction in response to VDZ induction therapy, while *MADCAM1* rs1555716175 was associated with optimal CRP reduction. Anti-drug antibody-related *HLA-DRB1*, *DQB1*, and *DQA1* alleles were not associated with VDZ response. **Conclusions.** This is a pioneering pharmacogenomics study on the VDZ direct therapeutic targets in bio-naïve CD patients, which opens the door for future research.

## 1. Introduction

Nowadays, medicine is shifting towards a more personalized approach. Precision medicine aims to tailor a patient’s treatment to his/her individual unique biological characteristics, and it is aligned with the biomedical model of health. Genomic data form the basis of precision medicine.

Pharmacogenomics (PGx) is the most prominent cornerstone of precision medicine. The efficacy and/or toxicity of a drug can be predicted based on the individual genetic profile of a patient, defining responders and non-responders to a drug. PGx completely changes the old therapeutic paradigm of “one dose fits all patients” and “trail-and-error” prescription, to a novel, personalized concept of “matching the right drug and the right dose to the specific genetic signature of the patient” [1]. Pharmacogenomic markers have been identified for several drugs, and genetic testing for these drugs has become available. Encouraging results of the application of PGx in clinical practice have moved research on pharmacogenomic markers to guide dosing and minimize the toxicity of a specific drug.

Crohn’s disease (CD) is an immune-mediated inflammatory disease that affects the gastrointestinal (GI) tract. It is considered that CD is the result of the complex interplay of several factors, including the genetic background and gut microbiome of a patient, environmental factors, and immune reactions at the GI mucosal surfaces [2]. Inflammation in patients with CD can affect all parts of the GI tract. The clinical manifestations and the course of CD are diverse. Disease relapses are frequent, and the quality of life in CD patients is often impaired. Numerous drugs are used to treat CD, although curative therapy is not yet available. The European Crohn’s and Colitis Organization (ECCO) has recommended guidelines for the use of different drugs for the induction and maintenance of remission in patients with CD [3]. The current management of CD is oriented towards the early control of inflammation. This approach includes the administration of biologics, such as monoclonal antibodies (mAbs), which may target at least two distinct pathological processes: they either block the effect of pro-inflammatory cytokines or prevent leukocyte migration to sites of inflammation [4]. Although the introduction of mAbs designed to inhibit tumor necrosis factor-α (TNF-α) has significantly improved CD treatment [5], primary non-response is observed in one-third of patients, and secondary loss of response and/or adverse events occur in almost one-half of patients [6].

**Figure 1:**
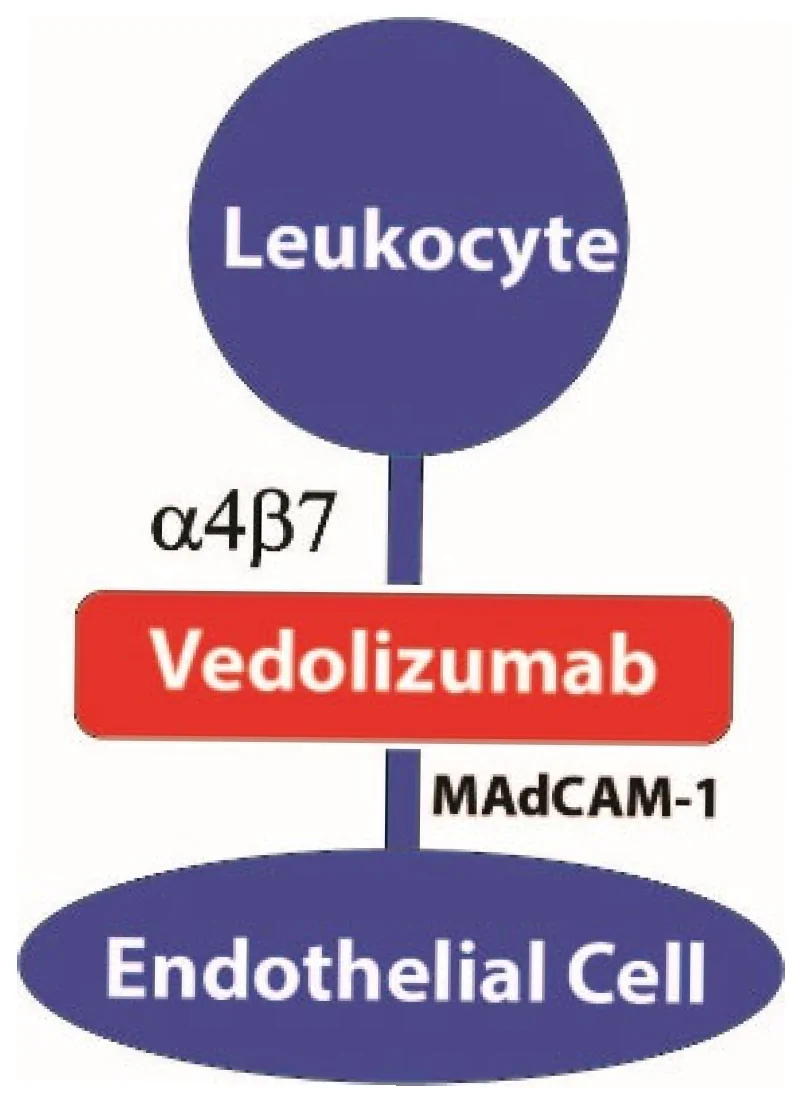
Schematic representation of the mechanism of vedolizumab action: prevention of interaction between α4β7 integrin on leukocytes and MAdCAM-1 molecule on the vascular endothelium.

One of the mAbs used in the treatment of CD after anti-TNF-α mAb is vedolizumab (VDZ). Vedolizumab is a recombinant humanized mAb. It acts against the α4β7 integrin, a cell-surface glycoprotein expressed on B and T lymphocytes. The protein α4β7 integrin interacts with cell adhesion molecules on the vascular endothelium, mucosal addressin-cell adhesion molecule 1 (MAdCAM-1), enabling the infiltration of the circulating leukocytes into inflamed intestinal tissues [7]. VDZ prevents interaction between α4β7 integrin and MAdCAM-1, thus avoiding the major pathogenic mechanism in CD [8] (Figure 1).

The efficacy of VDZ is limited to a subpopulation of patients with CD [9]. One possible explanation could be the individual pharmacogenomic profile.

There is a lack of data in the available literature regarding genetic markers of poor vedolizumab response [10,11]. Therefore, our research was focused on the variants in the genes encoding molecules involved in biological pathways targeted by the VDZ, such as the *ITGA4* gene, encoding the α4 subunit of integrin α4β7, the *ITGB7* gene, encoding the β7 subunit of integrin α4β7, and the *MADCAM1* gene, encoding MAdCAM-1 protein on the vascular endothelium, and their association with VDZ response in CD patients. Additionally, taking into consideration the importance of HLA (Human Leukocyte Antigen) genotyping in CD pathogenesis, as well as for prediction of adverse drug reactions (ADRs), we decided to expand our research on these genes, as potential pharmacogenomic markers of VDZ efficacy in CD [12,13,14]. Moreover, immunogenicity with formation of anti-drug antibodies (ADA) to biologics is an important reason for treatment failure in inflammatory bowel disease (IBD) [15], and it has been shown that ADA could be associated with specific HLA alleles.

## 2. Materials and Methods

### 2.1. Patients

Patients diagnosed with Crohn’s disease were enrolled from 2023 to 2025 at three referral IBD centers in Serbia. All 63 patients were treated with VDZ as a first-line advanced therapy. They were followed up, and data were analyzed after completion of the 14-week induction period. Vedolizumab was administered as an intravenous infusion (300 mg) at weeks 0, 2, 6, and 14 during the induction phase. In some patients, an additional infusion was administered at week 10 based on clinical indications. After 14 weeks of treatment, clinical and biochemical outcomes were assessed. Clinical response to VDZ treatment was estimated as follows: (1—based on the applied maintenance treatment protocol prescribed after the induction phase in week 14—good (therapy administrated every 8 weeks—standard protocol) or partial (therapy administrated every 4 weeks—optimized protocol), (2—based on the level of C-reactive protein evaluated at week 14)—good, (CRP ≤ 10 mg/L) or poor (CRP > 10 mg/L).

At week 14, the clinical response was assessed by the treating IBD specialist based on changes in abdominal pain and diarrhea/stool frequency, corresponding to the two patient-reported symptom domains included in PRO2 (2-item Patient-Reported Outcome). A good clinical response was defined as resolution of abdominal pain and diarrhea, whereas a partial clinical response was defined as an improvement in these symptoms compared with the baseline, with residual abdominal pain and/or diarrhea still present. Patients with a good clinical response continued standard VDZ maintenance therapy every 8 weeks, whereas patients with a partial response underwent early dose optimization to every 4 weeks [16].

Biochemical response was assessed using serum CRP levels at week 14. CRP was selected as an objective and standardized biomarker routinely used for monitoring inflammatory activity in CD. Patients were classified according to non-elevated versus elevated week-14 CRP values (≤10 mg/L versus >10 mg/L). Fecal calprotectin was evaluated as a secondary biochemical marker. For patients with CRP > 10 mg/L, clinical records were reviewed for potential alternative causes of CRP elevation, including active infection, acute intercurrent inflammatory conditions, clinically active extraintestinal manifestations, and other non-IBD-related inflammatory conditions. The cutoff of 10 mg/L corresponded to the upper limit of the reference range used by participating laboratories [17].

The study was conducted in accordance with the ethical standards of the Declaration of Helsinki, and approved by the Ethics Board, Clinical Centre of Serbia, on 28 February 2020, Prot. no. 432/7 and on 13 October 2025, Prot. No.1600/31. Ethical approvals were also obtained from the Ethics Board of University Clinical Center of Vojvodina (23 October 2025, Prot.no. 00-378) and the Ethics Board of University Hospital Medical Center “Zvezdara” (7 November 2025, Prot no. 05/11/2025). Written informed consent was obtained from all participants prior to inclusion in the study.

### 2.2. Genomic Analysis and Data Processing

Blood samples were collected from patients with CD for DNA isolation and whole exome sequencing (WES) analysis. Whole exome sequencing was performed using DNA Prep with Exome 2.5 Enrichment (Illumina, San Diego, CA, USA), and analysis was performed on Illumina NextSeq2000, following the manufacturer’s recommendations.

Sequencing raw data were processed following GATK best practices [18]. Variant annotation was performed using Variant Effect Predictor (VEP) version 113, which annotates genomic variants to determine their functional impact on genes and transcripts. Software prediction tools, such as MetaRNN, PROVEAN, and SIFT4G/LoF (accessed through the VEP’s plugin dbNSFP—release 4.7a), were used to predict the functionality of the variants, which were denoted as damaging, deleterious, tolerated, neutral, or loss-of-function (LoF), depending on the applied tool [19]. HLA genotypes were called using the HLA-HD tool, specifically optimized for these highly polymorphic regions [20]. Genetic variants that alter the protein sequence (missense, nonsense, splice-altering, insertion, deletion, and frameshift) from the three genes *ITGA4*, *ITGB7*, and *MADCAMl* were extracted and associated with the VDZ response. The same association was performed with HLA genotypes.

The association between genetic variants and VDZ response was analyzed using Fisher’s exact test. Additionally, for each genetic variant, a series of separate logistic regression models, each adjusting for one clinical covariate at a time—age, disease localization, or disease behavior was fitted to test if the genetic variants of interest are independent predictors of treatment outcome. For each model, odds ratio (OR) and its 95% confidence interval (CI), raw *p*-values, and *p*-values adjusted for multiple testing were reported. For the multiple correction method, the Benjamini–Hochberg method was applied. The threshold for statistical significance was set at *p* < 0.05. All statistical analyses were performed using R software (version 4.5.2).

## 3. Results

A total of 63 individuals with CD were enrolled in this study. The distribution of their demographic and clinical characteristics between responder groups is shown in Table 1. No differences in the demographic and major clinical characteristics were observed between patients with standard/optimized protocol and elevated/non-elevated CRP levels at week 14. Among nine patients with CRP > 10 mg/L at week 14, review of the available medical records did not identify either an active infection, an acute intercurrent inflammatory event, a clinically active extraintestinal inflammatory manifestation, or another evident non-IBD-related cause of CRP elevation.

After inspecting genetic variants in the *ITGA4*, *ITGB7*, and *MADCAM1* genes, a total of 10 genetic variants that alter protein sequence were selected, of which 7 were missense variants and 3 were insertions (1 in-frame and 2 frameshift). Most variants (*n* = 6) were of low frequency (MAF < 10%).

In this study, after induction therapy, 32 patients were treated with the standard protocol, and 31 patients were treated with the optimized maintenance protocol.

There were no statistically significant associations between selected genetic variants and response to VDZ therapy, defined by standard (good response) or optimized maintenance protocols (partial response) (Table 2).

The associations of genetic variants were evaluated in groups with a good biochemical response based on CRP level at week 14 of vedolizumab therapy (CRP ≤ 10 mg/L) or poor biochemical response (CRP > 10 mg/L) at week 14 of vedolizumab therapy. There were 54 patients with good biochemical response, and 9 with poor biochemical response. The genetic variant *MADCAM1* rs758941486 was associated with poor CRP reduction in response to VDZ therapy (*p* = 0.002), whereas *MADCAM1* rs1555716175 was associated with optimal CRP reduction (*p* = 0.008), using Fisher’s exact test (Table 2).

Next, the association of each variant with treatment response defined as CRP > 10 mg/L at week 14 was tested together with relevant covariates, such as age, disease localization, and disease behavior, in a series of multivariable logistic regression models. Each variant was thus assessed in three separately adjusted models, and the results of each are presented and identified by the covariate included (Table 3). This approach was chosen to evaluate the independence of each genetic association from individual clinical factors while respecting the limited number of outcome events. Our results showed that the *MADCAM1* rs758941486 variant was significantly associated with treatment response, independent of age, location, and behavior after multiple testing correction (*p* = 0.0075, *p* = 0.0085, *p* = 0.0157, respectively) (Table 3). *MADCAM1* rs1555716175 was also a predictor of treatment response, independent of age (*p* = 0.0435), while controlled for localization and behavior, it showed a statistical trend (*p* = 0.0788, *p* = 0.0924), after multiple correction adjustment.

Fecal calprotectin analysis did not show a statistically significant association with the investigated genetic variants. The absence of a significant association with fecal calprotectin may partly reflect the heterogeneity of the cohort regarding disease location and anatomical extent, since fecal calprotectin levels and optimal thresholds vary across isolated ileal, ileocolonic, and colonic CD and may be lower in limited or proximal small-bowel inflammation.

Next, we analyzed *HLA* alleles previously recognized as associated with anti-drug antibody (ADA) development, such as HLA-DRB1*03, HLA-DRB1*11, HLA-DQA1*05, and HLA-DQB1*02. Therapy response at week 14 (standard/optimized protocol or CRP level ≤ or >10 mg/dL) was not associated with HLA alleles related to ADA development in a studied group of CD patients (Table 4).

## 4. Discussion

Inadequate response is observed in approximately half of patients with CD treated with VDZ [21]. In these patients, a dose escalation by shortening the dosing interval to every 4 weeks during maintenance therapy was proven effective (optimized maintenance protocol) [22,23]. The same trend was observed in our study (standard maintenance protocol was applied in 32 patients and optimized maintenance protocol in 31 patients). This evidence clearly indicates that there are differences among patients in response to VDZ. Only a few studies have been conducted to identify pre-treatment predictors of response/non-response to VDZ, especially in CD bio-naïve patients [24,25].

However, none of these studies have resulted in actual predictive markers. For example, although differences between patients in gene expression profiles after VDZ therapy have been detected, none of these differentially expressed genes could be used as a predictive marker of treatment outcome [26,27]. However, some studies have reported promising results regarding predictive markers of response to VDZ. In one comprehensive study, subpopulations of biologic-naïve Crohn’s disease patients who would have a greater response to treatment with VDZ were identified, and the stratification of patients in certain subgroups was proposed [28]. In addition, the composition of the intestinal microbiome has been shown to be a potential predictive marker of VDZ response [29].

We hypothesized that the individual pharmacogenomic profile of patients could be useful in predicting VDZ treatment outcomes. Regarding VDZ, only a limited number of pharmacogenomic studies are available. In a study focused only on IBD-associated genes, variants in some of them were associated with either VDZ responders or non-responders. The rare variant analysis prioritized *NOD2*, *IL23*, *IL10*, *IL27*, and *TRAF1* genes in non-responders, and *CARD9*, *TYK2*, *IL4*, and *NLRP1* genes in responders. However, in this study, only 16 patients with IBD, including 4 with CD and 12 with ulcerative colitis, were analyzed [30]. In another comprehensive study using exome-based rare-variant analysis, it was shown that VDZ response was associated with genes involved in pathways related to membrane transport (ABC-family proteins, ion channels), L1–ankyrin interactions, bile acid recycling, and pathways involving MET signaling. In the same study, it was proposed that gene variants in pro-inflammatory lipid signaling and immune cell trafficking genes (e.g., *PIK3CG*, *CYP4F2*, *PLA2R1*), as well as variants in fatty acid oxidation and detoxification genes (e.g., *ACADM*, *CYP1A1*, *ALDH3A2*, *DECR1*, *MMUT*) could be used in a gene panel for stratification of patients with CD in an effort to optimize and personalize VDZ treatment [11].

In the context of a limited understanding of individual pharmacogenomic profiles of CD patients, in this study we have focused on the analysis of potential pharmacogenes involved in biological pathways directly targeted by the VDZ, such as *ITGA4* gene, encoding α4 subunit of integrin α4β7, *ITGB7* gene, encoding β7 subunit of integrin α4β7, and *MADCAM1* gene, encoding MAdCAM-1 protein, a cell adhesion molecule mainly expressed on endothelial cells of venules in mucosal tissues, such as the intestinal lamina propria and Peyer’s patches [31].

In our study, two variants in the *MADCAM1* gene were shown to be relevant for patients’ response to VDZ, defined by CRP > 10 mg/L at week 14 of VDZ therapy.

The first variant, *MADCAM1* rs758941486, is a loss-of-function variant with high confidence predicted by the SIFT4G algorithm. It represents an insertion at the position between codon 699 and 700, resulting in a frameshift that leads to an amino acid change from aspartic acid to asparagine at position 234, followed by termination of MAdCAM-1 protein synthesis after 77 base pairs (MADCAM1(NM_130760.3): c.699_700insAACACCACCTCCCC, p.(Asp234AsnfsTer77). It is shown that the genetic variant *MADCAM1* rs758941486 was associated with poor CRP reduction in response to VDZ therapy.

The other variant in the *MADCAM1* gene, a potential pharmacogenomic marker for VDZ response, is *MADCAM1* rs1555716175. It represents an insertion at the position between codon 717 and 718, resulting in an amino acid change from glutamic acid to serine at position 239, followed by insertion of 8 amino acids (c.717_718insCCTCCCAACACCACCTCCCCGGAG, p.(Glu239_Ser240insProProAsnThrThrSerProGlu)). It was associated with adequate CRP reduction.

VDZ interacts directly with integrin α4β7, and it is challenging to associate MAdCAM-1 protein dysfunction with poor or good therapeutic response to VDZ. This could be explained by the fact that the structural change of the MAdCAM-1 protein could disrupt its typical role in gut immunity through B-cell and memory T cell homing from the bloodstream.

One study demonstrated that the role of MAdCAM-1 in leukocyte adhesion is particularly important for VDZ response. It has been shown that T cell adhesion to MAdCAM-1 protein correlates with clinical response to VDZ treatment [32]. CD4+ T cells were isolated from the peripheral blood of patients treated with VDZ. In in vitro experiments, adhesion of those T cells to MAdCAM-1 protein was investigated, and it was shown that variable adhesion was feasible to predict VDZ response before treatment initiation [32]. To support the role of MAdCAM-1 functionality in the response to VDZ treatment, it was reported that differential levels of CD4+ T cell adhesion to MAdCAM-1 are most likely not a result of differential α4β7 integrin expression, but rather a differential intrinsic functionality of this pathway in responders versus non-responders [33].

There are two possible explanations for the change in MAdCAM-1 protein capacity in the adhesion of lymphocytes, which is caused by the variants that we have detected in our cohort of CD patients.

The first is directly correlated with the MAdCAM-1 protein adhesion capacity. Namely, MAdCAM-1 is a ligand not only for integrin α4β7 [7], but also for L-selectin, a cell surface C-type lectin, which contributes to lymphocyte homing to Peyer’s patches and to leukocyte interactions with inflamed venules. MAdCAM-1 protein is essential for L-selectin-mediated lymphocyte rolling and integrin-mediated adhesion [34], and L-selectin may be a modulating factor in the biological pathway involved in VDZ efficacy. Both variants shown to be potential pharmacogenomic markers of VDZ response in our cohort of CD patients are in the mucin-like domain of the MAdCAM-1 protein, which encompasses the protein region from amino acid 226 to amino acid 317. This region is responsible for binding L-selectin, which takes part in facilitating initial tethering and rolling of leukocytes. However, it is shown that isoform 2 of the MAdCAM-1 protein, characterized by the absence of the mucin-like domain (lacking amino acids 223–334), is specialized in supporting integrin α4β7-dependent adhesion strengthening, independent of L-selectin binding [31,35]. Consequently, the variant *MADCAM1* rs758941486, which results in a truncated MAdCAM-1 protein lacking a part of the mucin-like domain, could produce the effect of strengthening α4β7-integrin binding, presuming that VDZ does not block all α4β7-integrin receptor molecules. This result facilitates the migration of lymphocytes into the intestinal tissue, and the effect of VDZ is impaired.

The other hypothesis considers the entire endothelial environment of the intestinal tissue. We presume that variants detected in the *MADCAM1* gene could change the protein conformation and thus disrupt the functional organization of the vascular endothelium of the intestine. This could lead to strengthening or decreasing/weakening/failing the interactions of other integrins with other molecules with key overlapping functions related to cell adhesion and migration within the intestinal vasculature, such as VCAM-1 and fibronectin [36]. It is known that the pathway involving α4β1 integrin represents an alternative pathway for lymphocyte gut homing when α4β7 is blocked [37]. Therefore, the variant *MADCAM1* rs1555716175 could lead to conformational changes in the MAdCAM-1 protein, enabling crucial changes in the biological pathways that mediate leukocyte extravasation into inflamed intestinal tissues. This variant could be a positive prognostic marker for the response to VDZ treatment in patients with CD.

In both cases, the variants in the *MADCAM1* gene should not only be investigated independently as pharmacogenomic markers of response to VDZ but also as genetic markers that could influence the course of CD.

Inherited genetic variations in HLA molecules are known to play a role in ADA development, given their function in presenting peptide antigens to T cells. For example, the HLA-DQA1*05 allele was associated with ADA development in patients with Crohn’s disease treated with infliximab or adalimumab [38]. In our study, ADA-related *HLA-DRB1*, *DQB1* and *DQA1* alleles were not associated with VDZ response. This is in concordance with the results of the study in which it was shown that the HLA-DQA1*05 allele (rs2097432) was not associated with increased immunogenicity for VDZ [39].

This study has several limitations. Although it was conducted using a powerful methodology, WES, which enabled the detection of a significant number of variants in the coding region of selected genes, as well as different HLA alleles, its limitations, however, impacted the results. A more comprehensive (whole-genome sequencing) and hypothesis-free approach should be applied that would take into account rare variant burden analysis to decipher potential genomic determinants of poor/partial vedolizumab response. In addition, the hypotheses regarding the effect of conformational or mucin-like domain alterations in the MAdCAM-1 protein should be confirmed by in vitro functional studies. Furthermore, analysis of *MADCAM1* gene expression could be useful as it is significantly upregulated during active inflammation in CD. Since other biological pathways, such as pro-inflammatory cytokines, specifically TNF-α and IL-1β, and the NF-κB pathway, could interfere with the pathway that we investigated, the analysis of these factors should be included in the prediction of VDZ response in patients with CD [40].

The main limitation of this study is its small sample size, which is particularly relevant if variants are of low frequency, influencing the statistical power of the study. Most variants found in the selected genes are rare, and their associations could not be evaluated without a larger patient cohort. Therefore, this study should be interpreted as exploratory and hypothesis-generating, and the findings require confirmation in larger, independent cohorts.

Regarding the clinical and biochemical markers of therapy response, several issues have arisen that need to be considered with caution.

Thus, although the week-14 assessment incorporated the two principal patient-reported symptom domains included in PRO2—abdominal pain and stool frequency—a formal numerical PRO2 or HBI (Harvey Bradshaw Index) score was not calculated at this time point. Therefore, the clinical response classification should be regarded as a pragmatic symptom-based assessment used in routine practice rather than a formally validated disease activity endpoint [16].

CRP is a systemic and non-gut-specific inflammatory marker that has lower sensitivity for mucosal inflammation than fecal calprotectin or endoscopic assessment. Therefore, normal CRP values could not exclude persistent intestinal inflammation [41]. Although no evident alternative cause of CRP elevation was identified among the nine patients with elevated week-14 CRP, occult or unrecorded confounding conditions cannot be completely excluded. In addition, complete data on concomitant systemic corticosteroid use were not available for the entire cohort, precluding assessment of its potential influence on treatment response and representing a possible source of residual clinical confounding. Accordingly, the observed findings should be interpreted as exploratory associations with week 14 CRP status rather than as definitive predictors of comprehensive therapeutic response to VDZ.

This is one of the first studies in the field of pharmacogenomics and response to VDZ in bio-naïve CD patients. Two variants in the *MADCAM1* gene reported in this study are potential pharmacogenomic markers relevant to patients’ responses to VDZ. There is a strong biological rationale for further research on the VDZ direct therapeutic targets in CD patients in order to elucidate their pharmacogenomic potential. Novel markers pave the way to a precision-medicine approach in the treatment of CD, which could lead to the improvement of the management of this complex disease. However, in addition to pharmacogenomics, multi-omics approaches are indispensable for enabling the introduction of predictive markers of VDZ response in clinical practice.

## Figures and Tables

**Table 1:** Demographic and clinical characteristics of the CD group related to vedolizumab treatment response.

Demographic and Clinical Data	Standard Maintenance Protocol	Optimized Maintenance Protocol	Week 14 CRP ≤ 10 mg/L	Week 14 CRP > 10 mg/L
Number, *n*	32	31	54	9
Male, *n* (%)	11 (34.4)	16 (51.6)	25 (46.3)	2 (22.2)
Female, *n* (%)	21 (65.6)	15 (48.4)	29 (53.7)	7 (77.8)
Age at diagnosis *				
A1 < 17, *n* (%)	2 (6.3)	0 (0.0)	1 (1.9)	1 (12.5)
A2 17–40, *n* (%)	14 (43.7)	12 (40)	22 (40.7)	4 (50)
A3 > 40, *n* (%)	16 (50)	18 (60)	31 (57.4)	3 (37.5)
Disease localization *				
L1 (terminal ileum), *n* (%)	7 (21.9)	6 (19.4)	10 (18.5)	3 (33.3)
L2 (colon), *n* (%)	8 (25)	11 (35.5)	18 (33.3)	1 (11.1)
L3 (ileum and colon), *n* (%)	15 (46.8)	13 (41.9)	23 (42.6)	5 (55.6)
L4 (upper disease), *n* (%)	2 (6.3)	1 (3.2)	3 (5.6)	0 (0)
Disease behavior *				
B1 (inflammatory), *n* (%)	18 (56.2)	19 (61.3)	32 (59.3)	5 (55.6)
B2 (structuring), *n* (%)	10 (31.3)	6 (19.4)	15 (27.8)	1 (11.1)
B3 (penetrating), *n* (%)	3 (9.4)	5 (16.1)	5 (9.2)	3 (33.3)
Perianal disease				
yes, *n* (%)	1 (3.1)	1 (3.2)	2 (3.7)	0 (0)
no *n* (%)	31 (96.9%)	30 (96.8%)	52 (96.3%)	9 (100%)
Fecal calprotectin baseline, median [IQR]	283 [100–674]	453 [140–1096]	382 [131–986]	133 [52.5–291]
CRP baseline, median [IQR]	2.5 [0.738–4.68]	6.6 [1.85–11.2]	3.17 [1.08–6.95]	4.4 [1.4–15]
Immunomodulators				
yes, *n* (%)	10 (31.3)	12 (38.7)	35 (64.8)	6 (66.7)
no, *n* (%)	22 (68.7)	19 (61.3)	19 (35.2)	3 (33.3)
Dose administration at week 10				
Yes, *n* (%)	28 (87.5)	28 (90.3)	48 (88.9)	8 (88.9)
No, *n* (%)	4 (12.5)	3 (9.7)	6 (11.1)	1 (11.1)

* Disease characteristics were presented according to Montreal classification. Fisher’s exact test for discrete and Mann-Whitney test for continuous variables were used to test differences in distribution of demographic and clinical factors between standard/optimized and CRP non-elevated/elevated level at week 14 groups. No statistically significant difference was found among groups.

**Table 2:** Association analysis between VDZ CD patients’ response and selected genetic variants.

Gene	Position	Variant	Variant Type	Variant Effect Prediction Tools (MetaRNN, PROVEAN, SIFT4G/LoF)	Standard Maintenance Protocol *n* = 32		Optimized Maintenance Protocol *n* = 31		*p* Val	Week 14 CRP ≤ 10 mg/L, *n* = 54		Week 14 CRP > 10 mg/L, *n* = 9		*p* Val
					Ref/Ref, *n* (%)	Ref/Alt+ Alt/Alt, *n* (%)	Ref/Ref, *n* (%)	Ref/Alt+ Alt/Alt, *n* (%)		Ref/Ref, *n* (%)	Ref/Alt + Alt/Alt, *n* (%)	Ref/Ref, *n* (%)	Ref/Alt + Alt/Alt, *n* (%)	
*ITGA4*	chr2:181523468	c.2105A>T	Missense	D, N, D	31 (96.9%)	1 (3.1%)	31 (100%)	0 (0%)	1	53 (98.1%)	1 (1.9%)	9 (100%)	0 (0%)	1
*ITGA4*	chr2:181527311	rs55705823	Missense	T, N, D	31 (96.9%)	1 (3.1%)	31 (100%)	0 (0%)	1	53 (98.1%)	1 (1.9%)	9 (100%)	0 (0%)	1
*ITGA4*	chr2:181530618	rs1143676	Missense	T, N, T	4 (12.5%)	28 (87.5%)	6 (19.4%)	25 (80.6%)	0.51	9 (16.7%)	45 (83.3%)	1 (11.1%)	8 (88.9%)	1
*ITGB7*	chr12:53192471	rs11539433	Missense	T, N, T	32 (100%)	0 (0%)	29 (93.5%)	2 (6.5%)	0.24	53 (98.1%)	1 (1.9%)	8 (88.9%)	1 (11.1%)	0.27
*ITGB7*	chr12:53192752	rs61754162	Missense	T, D, T	32 (100%)	0 (0%)	30 (96.8%)	1 (3.2%)	0.49	54 (100%)	0 (0%)	8 (88.9%)	1 (11.1%)	0.14
*ITGB7*	chr12:53197863	rs7972747	Missense	T, N, T	31 (96.9%)	1 (3.1%)	31 (100%)	0 (0%)	1	52 (98.1%)	1 (1.9%)	9 (100%)	0 (0%)	1
*MADCAM1*	chr19:501697	rs758941486	Frameshift	HC	26 (81.2%)	6 (18.8%)	26 (83.9%)	5 (16.1%)	1	49 (90.7%)	5 (9.3%)	3 (33.3%)	6 (66.7%)	0.002
*MADCAM1*	chr19:501701	rs1555716175	inframe insertion	NA	11 (36.7%)	19 (63.3%)	12 (40%)	18 (60%)	1	16 (30.8%)	36 (69.2%)	7 (87.5%)	1 (12.5%)	0.008
*MADCAM1*	chr19:501742	rs753094852	frameshift variant	HC	32 (100%)	0 (0%)	30 (96.8%)	1 (3.2%)	0.49	53 (98.1%)	1 (1.9%)	9 (100%)	0 (0%)	1
*MADCAM1*	chr19:501900	rs3745925	missense	T, N, D	22 (68.7%)	10 (31.3%)	18 (58%)	13 (42%)	0.44	34 (63%)	20 (37%)	6 (66.7%)	3 (33.3%)	1

Annotation of the predicted effects: MetaRNN: D—Damaging, T—Tolerated; PROVEAN: D—Deleterious, N—Neutral; SIFT4G: D—Deleterious, T—Tolerated; LoF: HC—High Confidence Loss-of-function Variant, Ref—Reference Allele, Alt—Alternative allele.

**Table 3:** Multivariable logistic regression models of week-14 CRP response (CRP > 10 mg/L) using each genetic variant, adjusted for one of the clinically relevant covariates—age, disease localization, and disease behavior.

Genetic Variant		Covariables										
	Age				Localization				Behavior			
	*n*	OR [95%CI]	*p*	*p*adj	*n*	OR [95%CI]	*p*	*p*adj	*n*	OR [95%CI]	*p*	*p*adj
*ITGA4* chr2.181523468	63	NA *	0.995	0.9951	63	NA *	0.9967	1	63	NA *	0.997	0.997
*ITGA4* rs55705823	63	NA *	0.995	0.9951	63	NA *	1	1	63	NA *	0.997	0.997
*ITGA4* rs1143676	63	1.52 [0.23–30.21]	0.7087	0.9951	63	1.22 [0.13–11.78]	0.8641	1	63	1.58 [0.16–15.41]	0.6918	0.997
*ITGB7* rs11539433	63	6.01 [0.22–166.49]	0.2268	0.7937	63	4.37 [0.24–80.08]	0.3197	1	63	7.75 [0.40–149.71]	0.1753	0.6135
*ITGB7* rs61754162	63	NA *	0.9938	0.9951	63	NA *	0.9961	1	63	NA *	0.9963	0.997
*ITGB7* rs7972747	62	NA *	0.9951	0.9951	62	NA *	0.997	1	62	NA *	0.9951	0.997
*MADCAM1* rs758941486	63	20.71 [3.97–146.81]	**0.0007**	**0.0075**	63	20.83 [3.54–122.45]	**0.0008**	**0.0085**	63	15.13 [2.65–86.45]	**0.0022**	**0.0157**
*MADCAM1* rs1555716175	60	0.05 [0.002–0.34]	**0.0093**	**0.0435**	60	0.06 [0.006–0.60]	**0.0169**	**0.0788**	60	0.07 [0.007–0.66]	**0.0198**	**0.0924**
*MADCAM1* rs753094852	63	NA *	0.9949	0.9951	63	NA *	0.997	1	63	NA *	0.9968	0.997
*MADCAM1* rs3745925	63	0.86 [0.17–3.70]	0.8505	0.9951	63	0.73 [0.16–3.36]	0.6834	1	63	1.29 [0.26–6.47]	0.7581	0.997

For each variant, a logistic regression model was performed with one of the covariates (age, localization, or behavior of the disease). Odds ratios (OR), 95% confidence intervals (CI), and *p*-values were adjusted using the Benjamini–Hochberg false discovery rate (FDR) method for multiple testing correction. * NA-OR not estimable. For these variants, the minor allele was not present, or it was present in only one participant, producing complete or quasi-complete separation in the logistic model: the maximum-likelihood estimate did not converge to a finite value, and the 95% CI was unbounded. Therefore, the corresponding OR and CI were not reported. Bolded are significant *p*-values and *p*-values showing statistical trend (*p* < 0.1).

**Table 4:** *HLA* alleles related to ADA and response to VDZ induction therapy.

*HLA*			Standard Maintenance Protocol, *n* (%)	Optimized Maintenance Protocol, *n* (%)	*p*	CRP ≤ 10 mg/L Week 14, *n* (%)	CRP > 10 mg/L Week 14, *n* (%)	*p*
*DRB1*	*03	present	5 (15.6%)	8 (25.8%)	0.3649	10 (18.5%)	3 (33.3%)	0.376
		absent	27 (84.4%)	23 (74.2%)		44 (81.5%)	6 (66.7%)	
	*11	present	14 (43.7%)	9 (29%)	0.297	22 (40.7%)	1 (11.1%)	0.137
		absent	18 (56.3%)	22 (71%)		32 (59.3%)	8 (88.9%)	
*DQA1*	*05	present	18 (56.3%)	16 (51.6%)	0.8	30 (55.6%)	4 (44.4%)	0.7
		absent	14 (43.7%)	15 (48.4%)		24 (44.4%)	5 (55.6%)	
*DQB1*	*02	present	11 (34.4%)	12 (38.7%)	0.8	19 (35.2%)	4 (44.4%)	0.7
		absent	21 (65.6%)	19 (61.3%)		35 (64.8%)	5 (55.6%)	

Distribution of four *HLA* class II alleles—HLA-DRB1*03, HLA-DRB1*11, HLA-DQA1*05, and HLA-DQB1*02—in relation to the maintenance dosing protocol and CRP response at week 14. For each allele, carriers are shown as present versus absent, compared between patients on the standard and optimized maintenance protocols, and between CRP responders (CRP ≤ 10 mg/L) and non-responders (CRP > 10 mg/L) at week 14, with *p*-values from Fisher’s exact test.

## Data Availability

The raw data supporting the conclusions of this article will be made available by the authors on request.

## Abbreviations

The following abbreviations are used in this manuscript.

CD	Crohn’s disease
MAdCAM-1	Mucosal addressin-cell adhesion molecule 1
VDZ	Vedolizumab
HLA	Human Leukocyte Antigen
PGx	Pharmacogenomics
GI	Gastrointestinal tract
ECCO	The European Crohn’s and Colitis Organization
PRO2	2-item Patient-Reported Outcome
mAbs	Monoclonal antibodies
TNF-α	Tumor necrosis factor-α
ADRs	Adverse drug reactions
ADA	Anti-drug antibodies
IBD	Inflammatory bowel disease
WES	Whole exome sequencing
VEP	Variant Effect Predictor
LoF	Loss of function
OR	Odds ratio OR
CI	Confidence interval
